# Evaluation of an inpatient psychocardiological rehabilitation program in Austria: Psychosocial outcomes of a six-month cohort study

**DOI:** 10.1371/journal.pone.0322485

**Published:** 2025-05-27

**Authors:** Martin Matzka, Reinhard Lenz, Cathrin Köller-Janauschek, Maria Neubauer, Monika Mustak-Blagusz, Johann Mikl

**Affiliations:** 1 Department for Applied Research, Innovation and Medical Service Development, Pensionsversicherung, Vienna, Vienna, Austria; 2 Rehabilitation Center Felbring, Pensionsversicherung, Lower Austria, Muthmannsdorf, Austria; 3 DATA TECHNOLOGY Betriebsberatungs GmbH & Co KG, Vienna, Vienna, Austria; 4 Chief Medical Department, Pensionsversicherung, Vienna, Vienna, Austria; University of Zanjan, IRAN, ISLAMIC REPUBLIC OF

## Abstract

**Background:**

Resources for psychosocial care in conventional cardiologic settings are limited. To address this gap, the first inpatient psychocardiological rehabilitation program in Austria aimed at providing integrative rehabilitation for patients with concurrent heart and mental health conditions has been piloted. The six-week program is provided in two consecutive rehabilitation modules and focuses on psychological and psychotherapeutic interventions embedded within the key elements of conventional cardiac rehabilitation. The objective of this cohort study was to evaluate the psychosocial outcomes of the pilot program, with a particular emphasis on longer-term results.

**Methods:**

This study is a quantitative observational outcome evaluation based on patient-reported data collected at four consecutive time points during and six months after completing the two-part program. We conducted a repeated measures multivariate analysis of variance (MANOVA) with heart-focused anxiety (CAQ), global psychological distress (SCL-90®-S) and health-related quality of life (SF-12) as combined dependent variables. Time and social functioning (SAS-SR) on admission were included as explanatory variables. Ninety patients provided informed consent, finished the program and provided follow-up data.

**Results:**

Six months after rehabilitation, significant reductions were observed in patients’ heart-focused anxiety (η_p_^2^ =.144) and global psychological distress (η_p_^2^ =.057) as well as a significant increase in mental quality of life (η_p_^2^ =.137) compared to the admission values. Effect sizes expectedly decreased over time. Physical quality of life remained relatively constant over time and was the only outcome for which social integration on admission was not a significant prognostic factor.

**Conclusions:**

The results indicate sustainable reductions in psychosocial symptoms and improvements in health-related quality of life following the inpatient psychocardiological rehabilitation program in Austria. Targeted referral of patients to psychocardiological rehabilitation, individualized bio-psycho-social treatment plans and the provision of need-based, integrated aftercare are essential for achieving and sustaining optimal rehabilitation outcomes.

## Introduction

### Cardiovascular and mental health

Cardiovascular and mental health are interconnected. Mental health conditions such as anxiety disorders, adjustment disorders and depression, can be both a result of cardiovascular disease (i.e., psychological sequelae), as well as a causal factor of cardiac events and recurrent cardiac events [1–4]. Dysregulation of biological systems (e.g., alterations of stress responses), behavioral factors (e.g., adverse health behaviors, lower adherence to treatment) and social factors (e.g., lack of social support, socioeconomic disadvantages) are discussed as underlying mechanisms or contributing factors for this bidirectional link [1,3]. Consequently, comorbid mental health conditions are a major concern in cardiology.

Research shows that patient-reported anxiety, depression and health-related quality of life are independent predictors of one-year mortality and cardiac events after hospital discharge in patients with cardiovascular diseases [1]. Symptoms of anxiety and depression in these patients can fluctuate over time, both following acute cardiac events and in longer-term primary care settings, though psychological distress may persist or even worsen over time [5,6]. Heart-focused anxiety (HFA) can be an underlying factor or manifestation of distress commonly seen in both patients with cardiovascular diseases and those with mental health conditions [7]. HFA is characterized by increased attention to and monitoring of ambiguous heart-related sensations, avoidance behaviors to prevent their occurrence, and fears of their potentially life-threatening consequences [7,8]. As a specific fear of cardiac events and sensations, it is associated with a greater need for psychological support in cardiac rehabilitation and may negatively impact patients’ prognosis, their participation in social life [9], health behavior [10] and overall health-related quality of life [9,10].

### Cardiac rehabilitation and mental health

Despite mounting evidence on the role of mental health in cardiology, there is a notable lack of psychosocial screenings, psychiatric diagnostics and appropriate therapeutic measures in various cardiology settings, including rehabilitation [11]. Patients undergoing cardiac rehabilitation are at increased risk of experiencing clinically significant anxiety and depression, which can lead to lower treatment adherence, suboptimal treatment outcomes and diminished health-related quality of life [12,13]. Elevated symptoms of anxiety and depression may even persist five to ten years after cardiac rehabilitation [14]. These findings raise questions about the effectiveness of cardiac rehabilitation in addressing the complex psychosocial challenges and life circumstances that patients face. Social role impairment, or social functioning, is widely recognized as a vital indicator of mental health [15,16], and may contribute to differences in outcomes of rehabilitation. It encompasses the ability to form meaningful relationships, adapt to different social situations, and obtain support from others. Therefore, it is essential to consider social health needs carefully when planning medical treatment for patients with cardiovascular diseases [17,18].

Conventional rehabilitation for cardiac patients primarily focuses on physical exercise. When combined with various educational, lifestyle and/or psychological interventions, this approach is referred to as ”multimodal rehabilitation” [19]. In contrast, psychocardiological rehabilitation is a specific type of multimodal rehabilitation where psychological assessments and interventions are considered central and integrated, rather than optional, supplemental or isolated treatment modalities. Psychocardiology, as an emerging field in multiprofessional healthcare, examines the interactions between psychosocial and cardiovascular health and aims to provide integrated care across all cardiac care settings [20,21]. Currently, evidence supporting the effectiveness of inpatient psychocardiological rehabilitation is limited. Pilot studies conducted in Germany have reported significant reductions of heart-focused anxiety and psychological distress alongside significant improvements of mental health-related quality of life immediately following a 5-week inpatient rehabilitation program [22–24]. Furthermore, six months after the program, significant reductions in heart-focused anxiety and significant improvements in both mental and physical health-related quality of life were observed [22].

The first inpatient psychocardiological rehabilitation program in Austria was piloted from June 2019 to January 2024 at the Rehabilitation Center Felbring, which is operated by the Pensionsversicherung (PV). The present study started in May 2021 to evaluate the psychosocial outcomes of the pilot program, with a particular emphasis on longer-term outcomes observed six months post-rehabilitation. The results provide evidence on the effects of psychocardiological rehabilitation in inpatient settings and contributes to the refinement the program and its establishment as a standard psychocardiological rehabilitation service in Austria.

## Methods

### Study design and recruitment

**Fig 1:**
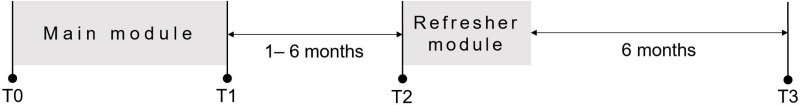
Consecutive time points of data collection.

The present study is a quantitative observational outcome evaluation based on patient-reported outcomes collected at four consecutive time points during and 6-months after a two-part inpatient rehabilitation program. Repeated assessments were conducted on admission (T0) and discharge (T1) from the four-week main rehabilitation module and on admission to the subsequent two-week inpatient refresher module (T2). These assessments were conducted routinely in the rehabilitation center. Six months after completing both modules, a follow-up survey was sent via mail (T3), marking the central endpoint of the present study (Fig 1).

The attending doctors invited patients admitted to psychocardiological rehabilitation at the Rehabilitation Center Felbring to participate in the evaluation study. Recruitment took place from May 10^th^, 2021 to October 27^th^, 2022. Patients received both verbal and written information about the study´s content and purpose and they voluntarily signed a consent form. Consistent with the primary admission criteria for the pilot phase of the program, the study included working-age patients between the ages of 18 and 64 years with cardiac (I10 - I95) and psychiatric diagnoses (F31 - F48) according to the International Classification of Diseases (ICD-10). Patients were excluded if they were unable to participate in rehabilitation due to acute and/or unstable psychiatric conditions or if they had severe neurocognitive or communicative deficits.

### Study population

**Fig 2:**
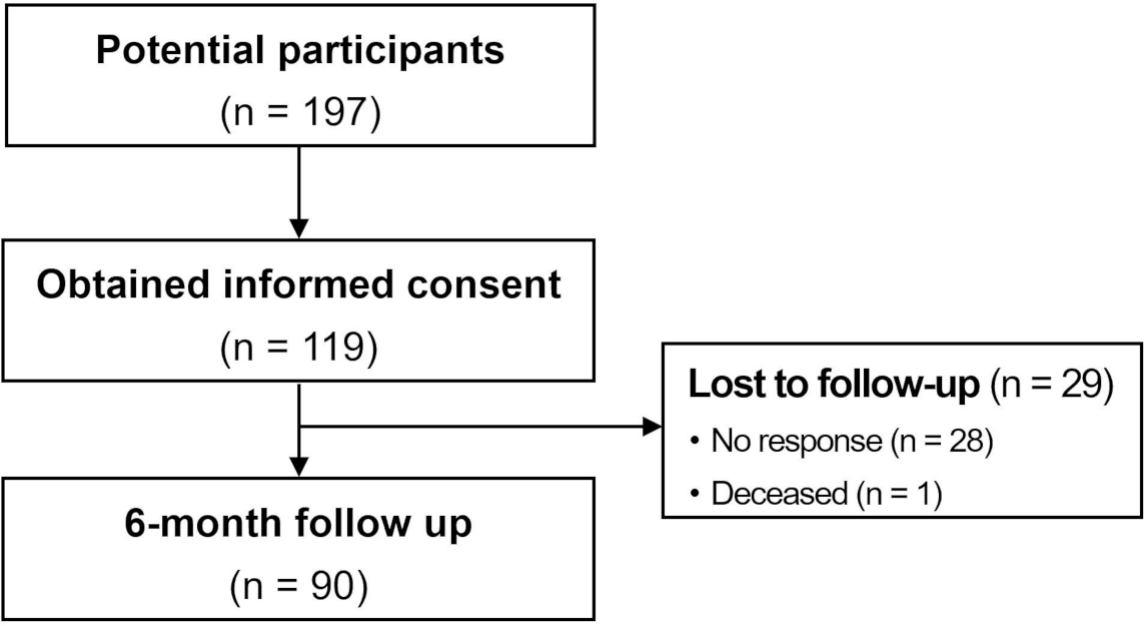
Flow chart of study sample.

Between May 2021 and October 2022, in total, 197 patients participated in the rehabilitation program. Of these, 119 consented to participate in the follow-up study. Ultimately, 90 patients (76%) completed the study by returning the follow-up questionnaire, which constitutes the sample for this research (Fig 2).

The study sample (n = 90) consists of 31 women (34.4%) and 59 men (65.6%). The average age of the participants at the time of admission was 53.6 ± 6.7 years, with a median age of 55 and a range of 20–64 years. Most participants were either living in a partnership or married (64.4%), had completed secondary or tertiary education (61.2%) and were employed (66.7%) or registered job seekers (30%) at the time of admission. The most common ICD-10 diagnoses leading to a referral for psychocardiological rehabilitation were ischaemic heart diseases (I20 - I25) and neurotic, stress-related and somatoform disorders (F40 - F48) (Table 1).

**Table 1:** Sociodemographic and medical characteristics of patients on admission (T0).

		N	%
Sex	Male	59	65.6%
	Female	31	34.4%
	Total	90	100%
Marital status	Single	11	12.2%
	Married/Domestic partnership	58	64.4%
	Divorced	19	21.1%
	Widowed	1	1.1%
	Total	89	98.9%
Highest educational level	Compulsory education (9 yrs.), with or without vocational training	34	37.8%
	Secondary education (10–13 yrs.)	32	35.6%
	Tertiary education	23	25.6%
	Total	89	98.9%
Employment status	Employed	60	66.7%
	Unemployed/ Registered job seekers	27	30.0%
	Other (e.g., temporary disability pension)	3	3.3%
	Total	90	100%
Cardiac diagnoses^a^	Ischaemic heart diseases (I21, I24, I25)	57	63.3%
	Other forms of heart disease (I35, I40, I42, I46 – I49, I50, I51)	25	27.8%
	Other cardiac diagnoses (I10, I26, I71, I72, I95)	8	8.9%
	Total	90	100%
Psychiatric diagnoses^b^	Neurotic, stress-related and somatoform disorders (F40, F41 – F43, F45)	48	53.3%
	Affective disorders (F31 – F33)	39	43.3%
	Affective disorders (F32, F33) in combination with neurotic and stress-related disorders (F41, F43)	3	3.3%
	Total	90	100%
Cardiovascular risk factors^c^	Hypercholesterolaemia/ Hyperlipidaemia (E78)	62	68.9%
	Essential hypertension (I10)	49	54.4%
	Smoking – past or present (F17)	34	37.8%
	Diabetes mellitus (E11)	23	25.6%
	Obesity (E66)	20	22.2%
	Hyperuricaemia (E79)	5	5.6%
		M	SD
Age	Age in years	53.6	6.7

N = sample size, % = frequency in the sample in percent, M = mean; SD = standard deviation; SAS-SR = Social Adjustment Scale – Self-report (total score).

a primary and ^b^ secondary ICD-10 diagnoses specified on the rehabilitation referral form, verified on admission. ^c^ risk factors in the patients’ medical history

### Intervention

All patients included in this study participated in a six-week inpatient psychocardiological rehabilitation, which consisted of two consecutive rehabilitation modules. First, patients underwent a four-week main rehabilitation module encompassing a total of 2400–3000 minutes of therapeutic interventions. Within one to six months following discharge, patients were readmitted to a two-week refresher module encompassing a total of 1200–1400 minutes of therapies. The aim of the refresher module is to reiterate and reinforce the therapeutic content of the main rehabilitation module and address problems and challenges that may have remained or arisen since the initial discharge. Examinations and diagnostics are performed on admission, discharge and readmission to provide a comprehensive assessment of the patients’ health status and health concerns (Table 2).

**Table 2:** Key diagnostics of psychocardiological rehabilitation.

**Physical and psychological diagnostics**
Electrocardiogram (ECG), exercise ECG, long-term ECG, echocardiogram, laboratory tests, cardiac radiography, psychiatric evaluation and psychological diagnostic assessments
**Optional diagnostics (if indicated)**
Long-term blood pressure monitoring, screening for sleep apnea, spiroergometry, stress-echocardiography

The psychocardiological rehabilitation program is based on a biopsychosocial understanding of health and ultimately aims to enhance patients’ participation in all areas of life. In the pilot phase of the program, it was offered primarily to patients of working age, but older patients may participate if medically indicated. Rehabilitation involves interdisciplinary case management and collaborative care provided by a team of cardiologists, psychiatrists, psychologists, physiotherapists, occupational therapists, dieticians and nurses. Each patient receives individually tailored therapeutic elements based on examination results, multiprofessional diagnostics, the patient’s performance level and the individual rehabilitation goals established with the patient. The rehabilitation program focuses on psychological and psychotherapeutic interventions in combination with essential components of conventional cardiac rehabilitation. Therapies are offered six days a week, from Monday through Saturday, between 7:30 a.m. and 5:00 p.m. (Table 3).

**Table 3:** Key components of psychocardiological rehabilitation.

Intervention	Delivery	Average frequency and duration^a^
Ergometer training	Individual	3 × 25min per week
Hiking/Nordic walking	Group (≤ 12 patients)	2 × 50min per week
Gymnastics/water gymnastics	Group (≤ 12 patients)	3 × 25min per week
Strength training	Group (≤ 12 patients)	3 × 25min per week
Psychotherapy	Individual	1 × 50min per week
Psychotherapy	Group (≤ 8 patients)	1 × 75min per week
Occupational therapy	Individual	3 × 50min (total)
Occupational therapy	Group (≤ 8 patients)	2 × 90min (total)
Patient education sessions	Group (≤ 12 patients)	2–4 × 25min (total)
Psychoeducational sessions	Group (≤ 25 patients)	3 × 90min (total)

^a^specified on a weekly basis or for the entire rehabilitation stay (i.e., 4+2 weeks)

Medical ward rounds occur once to twice a week. Patient education sessions address the topics of coronary heart disease, heart failure, diabetes, smoking cessation and relaxation techniques. Psychoeducational sessions cover the subjects of stress management, anxiety, depression and mindfulness. Additional rehabilitation services, such as individual physiotherapy sessions, massage therapy, art therapy, psychological counseling with relatives, cognitive training, biofeedback and smoking cessation programs, are provided as needed. As an integrative program, the rehabilitation’s recurrent and overarching themes are interactions of body, mind and social environment, strategies for coping with complex health conditions and health promoting behavior. At the conclusion of the rehabilitation program, all patients receive individualized aftercare recommendations.

### Assessments

Data on socio-demographic characteristics, medical history, and social functioning were routinely collected upon admission to rehabilitation (T0). Social functioning was evaluated with the 54-item Social Adjustment Scale – Self-report (SAS-SR). This scale measures both instrumental and expressive role performance over the past two weeks across several areas, including work, social and leisure activities, relationships with extended family, and roles as a marital partner, parent or family member. Respondents can skip items that are not relevant to their situation. Items are rated on a five-point scale. An overall mean score (ranging from 1 to 5) is calculated based on all completed items, with higher scores indicating greater impairment of social functioning [16,25].

The following assessment instruments were administered on admission (T0), discharge (T1), admission to the refresher module (T2) and the six-month postal follow-up (T3).

Heart-focused anxiety was assessed with the 17-item Cardiac Anxiety Questionnaire (CAQ). The items are rated on a five-point scale and address heart-related fears, avoidance behaviors and attention. A total score can be calculated, ranging from 0 to 4, with higher scores indicating higher heart-focused anxiety [7,8]. Global psychological distress was assessed with the Symptom-Checklist-90®-Standard (SCL-90®-S) [26], which is a revised instrument based on the original Symptom-Checklist-90 (SCL-90) [27]. This assessment assesses distress due to symptoms of anger/hostility, anxiety, depression, paranoid ideation, phobic anxiety, psychoticism, somatization, interpersonal sensitivity and obsessive-compulsive symptoms using 90 items. All items are rated on a five-point scale and aggregated in a global index of psychological distress, the Global Severity Index (GSI), which ranges from 0 to 4. Higher scores indicate greater psychological distress [26]. Lastly, health-related quality of life was assessed using the 12-item Short Form Health Survey (SF-12). This instrument evaluates functioning or impairment in various areas of life, the frequency of stressful mental symptoms and subjective overall health status. It yields separate, norm-based total scores for both physical (Physical Component Summary) and mental health-related quality of life (Mental Component Summary). The German standard sample (1994) with a mean of 50 ± 10 was used, with higher scores indicating higher quality of life [28,29].

All assessment instruments were provided in German language. Routine data (T0 - T2) was stored in a digital patient file system and exported for analysis. Data from the postal follow-up (T3) was added manually.

### Ethical considerations

The study protocol was submitted to the relevant ethics committee in Lower Austria (Austria), which concluded that there was no legal requirement for approval of this observational outcome evaluation. No interventions were conducted specifically for study purposes. The pilot program was implemented independently of the evaluation and adhered to the legal regulations in Austria. As the operator of the rehabilitation center Felbring, the Pensionsversicherung (PV) ensured that research ethics principles, such as informed consent and the protection of participants´ health, integrity, and data, were respected at all times. Participation in the study was voluntary, and refusal to participate or premature withdrawal did not affect the quality of patient care. Written informed consent was obtained from the patients.

### Statistical analysis

For the primary analysis, we performed a one-way repeated measures multivariate analysis of variance (MANOVA). The combined dependent variables were heart-focused anxiety, global psychological distress, and both physical and mental health-related quality of life. Time and impairment of social functioning were used as explanatory variables. Pillai’s trace was the main criterion for evaluating significance. Mauchly’s test of sphericity indicated that the assumption of sphericity was violated, which was addressed by applying the Greenhouse-Geisser correction. Partial eta squared (η_p_^2^) was calculated as effect size and interpreted as small (.0099 –.0587), medium (.0588 –.1378) and large (≥.1379) effects [30].

Post hoc analyses for the effect over time and analyses of subgroups with different levels of social functioning (tertiles) were performed by univariate analysis of variance, applying a Bonferroni correction for multiple testing. All contrasts were specified as simple contrasts, with the first group as reference. Additional insights into the dataset were obtained with correlational analyses and descriptive statistics. Missing data were not imputed. All analyses were performed using IBM SPSS Statistics, Version 28.0 (Armonk, NY, USA).

## Results

### Descriptive statistics

**Fig 3:**
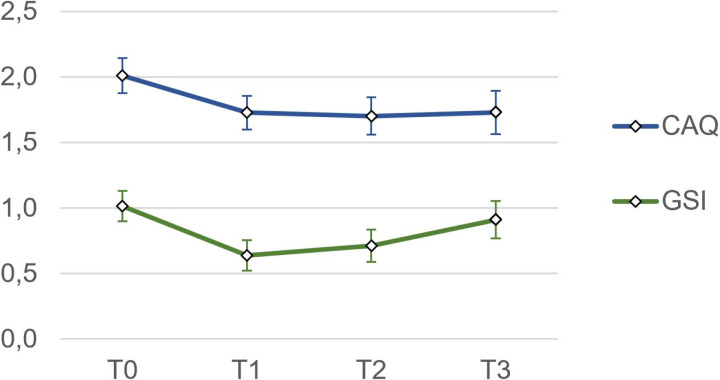
Trajectories of heart-focused anxiety and global psychological distress over time (means and 95% confidence interval). CAQ = Cardiac Anxiety Questionnaire total (heart-focused anxiety); GSI = Global Severity Index (SCL-90-S; global psychological distress).

**Fig 4:**
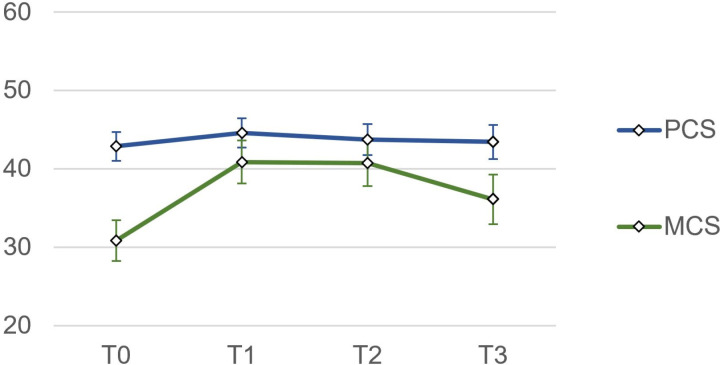
Trajectories of physical and mental health-related quality of life over time (means and 95% confidence interval). PCS = Physical Component Summary (SF-12; physical health-related quality of life); MCS = Mental Component Summary (SF-12; mental health-related quality of life).

Psychosocial outcomes were measured at four different time points: upon admission to (T0) and discharge from the main module (T1), upon readmission to the refresher module (T2), and six months after discharge from the refresher module (T3). Changes over time are illustrated in Figs 3 and 4, and the mean values can be found in S1 Table. The average timespan between discharge from the main rehabilitation module (T1) and admission to the refresher module (T2) was 84.9 ± 39.1 days, with a range of 34–183 days.

### Psychosocial outcomes by social functioning groups

Patients reported different levels of impairment in social functioning on admission, with a total mean score of 2.2 ± 0.6 on the SAS-SR (range 1.03–3.81). Patients with lower impairment (≤ 1.91), moderate impairment (> 1.91, ≤ 2.40) and higher impairment (> 2.4) differed in all psychosocial outcomes. For reference, see Table 4. In comparison with the other groups, patients with lower impairments of social functioning reported lower heart-focused anxiety and general psychological distress, along with higher physical and mental health-related quality of life during and after psychocardiological rehabilitation. In absolute terms, heart-focused anxiety and global psychological distress decreased most while quality of life improved the most between admission and follow-up for patients experiencing the highest impairment in social functioning.

**Table 4:** Psychosocial outcomes from admission to six-month follow-up in patients with low, moderate and high impairment of social functioning on admission.

	Admission (T0)			Discharge (T1)			Refresher module (T2)			Follow-up (T3)		
	N	M	SD	N	M	SD	N	M	SD	N	M	SD
**CAQ**												
Low SAS-SR	30	1.67	0.62	30	1.39	0.55	30	1.36	0.65	30	1.40	0.64
Moderate SAS-SR	31	2.09	0.53	31	1.82	0.54	31	1.76	0.62	31	1.95	0.81
High SAS-SR	29	2.28	0.61	29	1.98	0.63	29	2.00	0.65	29	1.84	0.83
**GSI**												
Low SAS-SR	30	0.53	0.26	30	0.29	0.22	30	0.28	0.22	30	0.48	0.40
Moderate SAS-SR	31	1.00	0.34	31	0.59	0.34	31	0.73	0.45	30	1.00	0.58
High SAS-SR	29	1.53	0.51	29	1.05	0.72	29	1.14	0.67	29	1.26	0.78
**PCS**												
Low SAS-SR	30	45.22	8.88	30	47.32	8.65	30	46.95	9.67	29	45.86	9.98
Moderate SAS-SR	31	43.10	9.45	31	42.95	8.56	31	43.99	9.62	31	41.08	9.67
High SAS-SR	29	40.16	7.35	29	43.52	9.31	29	40.11	8.25	27	43.52	10.57
**MCS**												
Low SAS-SR	30	41.38	9.51	30	49.20	8.81	30	50.95	9.42	29	45.96	12.69
Moderate SAS-SR	31	29.84	10.43	31	40.15	11.98	31	39.28	12.40	31	33.41	12.56
High SAS-SR	29	21.02	8.17	29	33.00	12.93	29	31.68	12.62	27	28.63	14.06

N = sample size; M = mean; SD = standard deviation; CAQ = Cardiac Anxiety Questionnaire total (heart-focused anxiety); GSI = Global Severity Index (SCL-90-S; global psychological distress); PCS = Physical Component Summary (SF-12; physical health-related quality of life); MCS = Mental Component Summary (SF-12; mental health-related quality of life); SAS-SR = Social Adjustment Scale – Self-report total (impairment of social functioning).

Correlations between social functioning and all psychosocial outcomes, as well as intercorrelations among outcomes, are detailed in S2 Table for reference. Social functioning was significantly correlated with heart-focused anxiety, global psychological distress and mental health-related quality of life at all time points (r =.35 to r =.76). In contrast, lower and not consistently significant correlations were observed between social functioning and physical health-related quality of life (r =.21 to r =.38).

### Primary analysis

In a first step, we checked the model assumptions of the repeated measures MANOVA. An explorative analysis showed that the normal distribution assumption could be accepted throughout the analysis groups. Mauchly’s test of sphericity revealed that the assumption of sphericity was violated for three of four indicators, prompting us to apply the Greenhouse-Geisser correction.

The results of the one-way repeated measures MANOVA demonstrated significant differences in the combined dependent variables (i.e., psychosocial outcomes) across time points (F(12, 72) = 10.796, p <.001, η_p_^2^ =.643, Pillai’s trace V =.643). Additionally, there were significant differences based on degrees of social functioning on admission (F(8, 162) = 6.782, p <.001, η_p_^2^ =.251, Pillai’s trace V =.502). No significant differences were found for the interaction between time and social functioning on admission (F(24, 146) = 1.034, p =.427, η_p_^2^ = 0.145, Pillai’s trace V =.291). We also conducted separate repeated measures MANOVAs using age, sex and psychiatric diagnoses groups (affective vs. neurotic disorders) as independent variables, which showed no significant differences in our combined outcome variables.

### Post-hoc univariate ANOVAs

Time was a significant predictor of heart-focused anxiety (F(2.374, 197.036) = 11.861, p <.001, η_p_^2^ =.125), global psychological distress (F(2.626, 217.993) = 32.937, p <.001, η_p_^2^ =.284), and mental health-related quality of life (F(3, 249) = 25.971, p <.001, η_p_^2^ =.238). Yet, time was not a significant predictor of physical health-related quality of life (F(2.499, 207.417) = 1.578, p =.203, η_p_^2^ =.019).

Patients‘ heart-focused anxiety and global psychological distress were consistently lower during and after rehabilitation than on admission. While effect sizes were large, they showed a decrease over time. For psychological distress, a small effect size remains at follow-up. In terms of physical quality of life, there was initially a large effect size (T0 - T1), but it subsequently decreases over time to a below small effect size. This indicates that the improvement in physical quality of life following the main rehabilitation module was temporary and not sustained up to the follow-up. The corresponding effect sizes for mental quality of life also decreased over time, but remained medium up to the follow-up assessment. This indicates that patients experienced an increase in mental quality of life during rehabilitation, and this improvement was largely sustained up to the six-months follow-up (Table 5).

**Table 5:** Test statistics of the within-subjects contrasts for all dependent variables with admission values as reference.

	F	df	p	η_p_^2^
**CAQ**				
T0-T1	31.638	(1, 83)	<.001	.276
T0-T2	21.198	(1, 83)	<.001	.203
T0-T3	13.922	(1, 83)	<.001	.144
**GSI**				
T0-T1	96.938	(1, 83)	<.001	.539
T0-T2	52.772	(1, 83)	<.001	.389
T0-T3	4.979	(1, 83)	.028	.057
**PCS**				
T0-T1	5.606	(1, 83)	.020	.063
T0-T2	2.552	(1, 83)	.114	.030
T0-T3	0.514	(1, 83)	.476	.006
**MCS**				
T0-T1	72.760	(1, 83)	<.001	.467
T0-T2	61.438	(1, 83)	<.001	.425
T0-T3	13.123	(1, 83)	<.001	.137

F = F-value; df = degrees of freedom (hypothesis, error), p = p-value; η_p_^2^ = partial eta squared; CAQ = Cardiac Anxiety Questionnaire total (heart-focused anxiety); GSI = Global Severity Index (SCL-90-S; global psychological distress); PCS = Physical Component Summary (SF-12; physical health-related quality of life); MCS = Mental Component Summary (SF-12; mental health-related quality of life).

Social functioning at the time of admission was a significant predictor of heart-focused anxiety (F(2, 83) = 8.674, p <.001, η_p_^2^ =.173), global psychological distress (F(2, 83) = 26.528, p <.001, η_p_^2^ =.390), and mental health-related quality of life (F(2, 83) = 28.754, p <.001, η_p_^2^ =.409). However, it did not significantly predict physical health-related quality of life (F(2, 83) = 2.846, p =.064, η_p_^2^ =.064).

### Post hoc analysis

**Fig 5:**
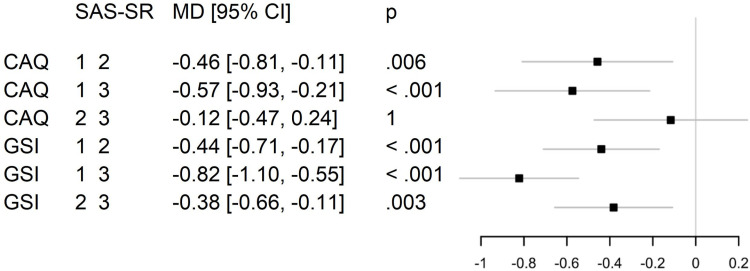
Mean differences in heart-focused anxiety and global psychological distress between patient groups with different levels of impairment in social functioning. MD = mean difference; CI = confidence interval; p = p-value; SAS-SR = Social Adjustment Scale – Self-report total, divided into groups (group 1 = lowest, 2 = moderate and 3 = highest impairment of social functioning); CAQ = Cardiac Anxiety Questionnaire total (heart-focused anxiety); GSI = Global Severity Index (SCL-90-S; global psychological distress).

**Fig 6:**
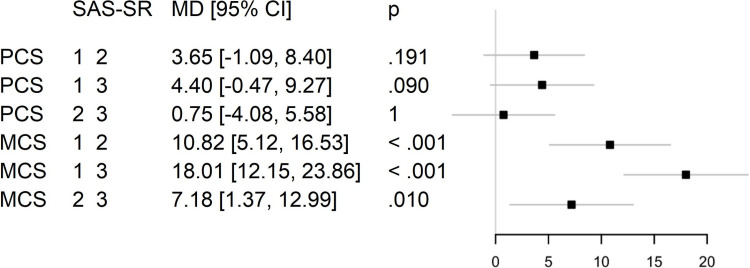
Mean differences in physical and mental health-related quality of life between patient groups with different levels of impairment in social functioning. MD = mean difference; CI = confidence interval; p = p-value; SAS-SR = Social Adjustment Scale – Self-report total, divided into groups (group 1 = lowest, 2 = moderate and 3 = highest impairment of social functioning); PCS = Physical Component Summary (SF-12; physical health-related quality of life); MCS = Mental Component Summary (SF-12; mental health-related quality of life).

Post hoc analysis with Bonferroni adjustment revealed that heart-focused anxiety and global psychological distress were significantly lower in patients with least impairment in social functioning compared to those with moderate and higher impairment. Mental health-related quality of life was significantly higher in patients with the least social impairments than in those with moderate and higher impairment. No statistically significant differences were found in physical health-related quality of life among the respective patient groups (Figs 5 and 6).

## Discussion

In this cohort study, we observed significant short and longer term reductions of patients’ heart-focused anxiety and global psychological distress, as well as improvements in mental health-related quality of life, six months after patients participated in inpatient psychocardiological rehabilitation. The initial effect sizes from admission to discharge from the main rehabilitation module (T0 - T1) were large for all four psychosocial study outcomes, but they decreased over time. The large effect sizes for changes of heart-focused anxiety and mental health-related quality of life were largely sustained at the six-month follow-up, while the longer term effect sizes for global psychological distress and physical health-related quality of life were reduced to small and below small, respectively. In summary, this suggests that patients undergoing psychocardiological rehabilitation are able to sustainably reduce both their global and heart-specific psychological distress while improving mental quality of life and maintaining their physical quality of life.

Over the course of rehabilitation (T0 - T2), global psychological distress decreased more than heart-focused anxiety, while the opposite was observed at the six-month follow-up. This suggests that integrative psychocardiological care addresses both the global and specific manifestations of patients’ psychological distress, but the impact varies over time. As a prominent therapeutic target of the condensed, six-week program, heart-focused anxiety was reduced more sustainably than global distress, indicating that patients may be coping better with their cardiac condition. Nonetheless, some patients may still require continuation and/or maintenance treatment after rehabilitation, especially given the risk of relapses or recurrences in patients with affective [31] and anxiety disorders [32]. These findings are largely consistent with a pilot study on inpatient psychocardiological rehabilitation in Germany, which reported significantly lower heart-related anxiety and non-significant reductions of psychological distress in patients at the six-month follow-up [22].

We also observed notable differences in our sample regarding the initial levels and subsequent development of patients’ physical and mental health-related quality of life. Both components of quality of life were rated below the average of the normative sample (50 ± 10), however, mental quality of life was markedly lower than physical quality of life on admission. Over time, the difference between the two components decreased. Physical quality of life remained relatively stable, while mental quality of life showed significant improvement. This finding slightly differs from the results of the German pilot study, which reported significant improvements in both components of quality of life six months after rehabilitation [22]. Differences in the study samples may provide one possible explanation for this discrepancy. Patients in our study had slightly higher physical quality of life and markedly lower mental quality of life on admission, which might have led to a different therapeutic focus. By the six-month follow-up, however, only marginal differences in quality of life between the two study samples remained [22].

We found that impairment in social functioning on admission to rehabilitation was a significant prognostic factor for heart-focused anxiety, global psychological distress and mental health-related quality of life. Social functioning most strongly correlated with psychological distress, but it also correlated significantly with mental quality of life and heart-focused anxiety at all assessment points. Its association with physical quality of life was weaker and not consistently significant over time. In summary, our analysis indicates that patients with greater impairment in social functioning on admission benefit from psychocardiological rehabilitation, but continue to exhibit more psychosocial symptoms and lower, especially mental, quality of life compared to patients with less social impairment. These findings highlight the importance of social functioning as an indicator for mental health, which should be considered in the treatment of patients with cardiovascular diseases [17,18]. Specifically, within the context of psychocardiological rehabilitation, high levels of social functioning may serve as a crucial psychosocial resource, while significantly impaired social functioning poses a risk for suboptimal patient-reported treatment outcomes.

### Limitations

The absence of a control group and the non-negligible loss to follow up of 29 patients (24.4%) are key limitations of this study. Patients lost to follow up were excluded from the analysis because the primary focus was on the long-term outcomes of rehabilitation. On average, these patients were notably younger than those in the final sample (48.38 ± 8.76), although there were no marked differences in terms of cardiac and psychiatric diagnoses. However, an analysis of the available data from time points T0 to T2 showed no substantial differences between the outcomes of the patients lost to follow-up and the final sample. Furthermore, to minimize the response burden, we chose not to conduct assessments on discharge from the refresher module and did not reassess social integration at follow-up, which could have provided additional insights.

## Conclusions

Considering the methodological limitations of this observational cohort study, we present our findings as indicators of sustainable psychosocial symptom reductions and improvements in health-related quality of life following an inpatient psychocardiological rehabilitation program in Austria. It is crucial to ensure targeted referral of patients to psychocardiological rehabilitation, implement individualized bio-psycho-social treatment plans and provide need-based, integrative aftercare. These steps are essential for achieving and maintaining optimal rehabilitation outcomes.

## Supporting information

S1 Table Psychosocial outcomes from admission to 6-month follow-up. N = sample size; M = mean; SD = standard deviation; CAQ = Cardiac Anxiety Questionnaire total (heart-focused anxiety); GSI = Global Severity Index (SCL-90-S; global psychological distress); PCS = Physical Component Summary (SF-12; physical health-related quality of life); MCS = Mental Component Summary (SF-12; mental health-related quality of life).
(DOCX)

S2 Table Correlations of impairment in social functioning and psychosocial outcomes (Pearson correlation coefficients). SAS-SR = Social Adjustment Scale – Self-report total (impairment of social functioning; T0); CAQ = Cardiac Anxiety Questionnaire total (heart-focused anxiety); GSI = Global Severity Index (SCL-90-S; global psychological distress); PCS = Physical Component Summary (SF-12; physical health-related quality of life); MCS = Mental Component Summary (SF-12; mental health-related quality of life); T0 = admission main module; T1 = discharge main module; T2 = admission refresher module; T3 = 6-month follow-up; * p <.05; ** p <.01; *** p <.001.
(DOCX)
