# Supplementary material for: Evaluation of an inpatient psychocardiological rehabilitation program in Austria: Psychosocial outcomes of a six-month cohort study
Source: PLoS One. 2025 May 27;20(5):e0322485. doi: 10.1371/journal.pone.0322485 (PMC12112417; doi:10.1371/journal.pone.0322485)
Supplement: S1 Table — N = sample size; M = mean; SD = standard deviation; CAQ = Cardiac Anxiety Questionnaire total (heart-focused anxiety); GSI = Global Severity Index (SCL-90-S; global psychological distress); PCS = Physical Component Summary (SF-12; physical health-related quality of life); MCS = Mental Component Summary (SF-12; mental health-related quality of life). (DOCX) [file pone.0322485.s001.docx]

**S1 Table. Psychosocial outcomes from admission to 6-month follow-up.**

|  | **Admission**  **(T0)** | | |  | **Discharge**  **(T1)** | | |  | **Refresher module (T2)** | | |  | **Follow-up**  **(T3)** | | |
| --- | --- | --- | --- | --- | --- | --- | --- | --- | --- | --- | --- | --- | --- | --- | --- |
|  | **N** | **M** | **SD** |  | **N** | **M** | **SD** |  | **N** | **M** | **SD** |  | **N** | **M** | **SD** |
| **CAQ** | 90 | 2.01 | 0.64 |  | 90 | 1.73 | 0.62 |  | 90 | 1.70 | 0.68 |  | 90 | 1.73 | 0.79 |
| **GSI** | 90 | 1.02 | 0.56 |  | 90 | 0.64 | 0.56 |  | 90 | 0.71 | 0.59 |  | 89 | 0.91 | 0.68 |
| **PCS** | 90 | 42.86 | 8.78 |  | 90 | 44.59 | 8.95 |  | 90 | 43.73 | 9.53 |  | 87 | 43.43 | 10.14 |
| **MCS** | 90 | 30.85 | 12.50 |  | 90 | 40.87 | 13.04 |  | 90 | 40.72 | 13.91 |  | 87 | 36.11 | 14.84 |

N = sample size; M = mean; SD = standard deviation; CAQ = Cardiac Anxiety Questionnaire total (heart-focused anxiety); GSI = Global severity scale (SCL-90-S; global psychological distress); PCS = Physical component summary (SF-12; physical health-related quality of life); MCS = Mental component summary (SF-12; mental health-related quality of life).
