# Supplementary material for: Evaluation of an inpatient psychocardiological rehabilitation program in Austria: Psychosocial outcomes of a six-month cohort study
Source: PLoS One. 2025 May 27;20(5):e0322485. doi: 10.1371/journal.pone.0322485 (PMC12112417; doi:10.1371/journal.pone.0322485)
Supplement: S2 Table — SAS-SR = Social Adjustment Scale – Self-report total (impairment of social functioning; T0); CAQ = Cardiac Anxiety Questionnaire total (heart-focused anxiety); GSI = Global Severity Index (SCL-90-S; global psychological distress); PCS = Physical Component Summary (SF-12; physical health-related quality of life); MCS = Mental Component Summary (SF-12; mental health-related quality of life); T0 = admission main module; T1 = discharge main module; T2 = admission refresher module; T3 = 6-month follow-up; * p <.05; ** p <.01; *** p <.001. (DOCX) [file pone.0322485.s002.docx]

|  | **SAS-SR** | **CAQ T0** | **CAQ T1** | **CAQ T2** | **CAQ T3** | **GSI T0** | **GSI T1** | **GSI T2** | **GSI T3** | **PCS T0** | **PCS T1** | **PCS T2** | **PCS T3** | **MCS T0** | **MCS T1** | **MCS T2** | **MCS T3** |
| --- | --- | --- | --- | --- | --- | --- | --- | --- | --- | --- | --- | --- | --- | --- | --- | --- | --- |
| **SAS-SR** | - |  |  |  |  |  |  |  |  |  |  |  |  |  |  |  |  |
| **CAQ T0** | ,423*** | - |  |  |  |  |  |  |  |  |  |  |  |  |  |  |  |
| **CAQ T1** | ,417*** | ,710*** | - |  |  |  |  |  |  |  |  |  |  |  |  |  |  |
| **CAQ T2** | ,448*** | ,574*** | ,771*** | - |  |  |  |  |  |  |  |  |  |  |  |  |  |
| **CAQ T3** | ,347** | ,508*** | ,715*** | ,738*** | - |  |  |  |  |  |  |  |  |  |  |  |  |
| **GSI T0** | ,757*** | ,451*** | ,494*** | ,460*** | ,330** | - |  |  |  |  |  |  |  |  |  |  |  |
| **GSI T1** | ,610*** | ,361*** | ,594*** | ,530*** | ,456*** | ,797*** | - |  |  |  |  |  |  |  |  |  |  |
| **GSI T2** | ,608*** | ,250* | ,471*** | ,531*** | ,418*** | ,786*** | ,867*** | - |  |  |  |  |  |  |  |  |  |
| **GSI T3** | ,557*** | ,360** | ,599*** | ,591*** | ,683*** | ,727*** | ,797*** | ,795*** | - |  |  |  |  |  |  |  |  |
| **PCS T0** | -,286** | -,299** | -,322** | -,360*** | -,277** | -,357** | -,315** | -,350** | -,329** | - |  |  |  |  |  |  |  |
| **PCS T1** | -,244* | -,267* | -,459*** | -,423*** | -,370*** | -,395*** | -,451*** | -,410*** | -,396*** | ,574*** | - |  |  |  |  |  |  |
| **PCS T2** | -,378*** | -,197 | -,415*** | -,523*** | -,389*** | -,493*** | -,526*** | -,571*** | -,476*** | ,671*** | ,607*** | - |  |  |  |  |  |
| **PCS T3** | -,209 | -,233* | -,379*** | -,503*** | -,641*** | -,385*** | -,415*** | -,453*** | -,588*** | ,459*** | ,316** | ,561*** | - |  |  |  |  |
| **MCS T0** | -,587*** | -,341** | -,371*** | -,363*** | -,186 | -,664*** | -,516*** | -,548*** | -,526*** | ,235* | ,330** | ,274** | ,193 | - |  |  |  |
| **MCS T1** | -,481*** | -,316** | -,568*** | -,471*** | -,351** | -,579*** | -,772*** | -,639*** | -,634*** | ,249* | ,392*** | ,378*** | ,293** | ,649*** | - |  |  |
| **MCS T2** | -,505*** | -,174 | -,372*** | -,487*** | -,323** | -,549*** | -,632*** | -,772*** | -,610*** | ,237* | ,333** | ,348** | ,331** | ,640*** | ,630*** | - |  |
| **MCS T3** | -,458*** | -,299** | -,474*** | -,470*** | -,507*** | -,488*** | -,557*** | -,594*** | -,759*** | ,278** | ,414*** | ,302** | ,316** | ,553*** | ,665*** | ,641*** | - |

**S2 Table.** **Correlations of impairment in social functioning and psychosocial outcomes (Pearson correlation coefficients).**

SAS-SR = Social Adjustment Scale – Self-report total (impairment of social functioning; T0); CAQ = Cardiac Anxiety Questionnaire total (heart-focused anxiety); GSI = Global severity scale (SCL-90-S; global psychological distress); PCS = Physical component summary (SF-12; physical health-related quality of life); MCS = Mental component summary (SF-12; mental health-related quality of life); T0 = admission main module; T1 = discharge main module; T2 = admission refresher module; T3 = 6-month follow-up; * p < .05; ** p < .01; *** p < .001.
